# Reviewer Acknowledgment

**DOI:** 10.1002/kjm2.12789

**Published:** 2023-11-20

Consistent high‐quality of papers published in “Kaohsiung Journal of Medical Sciences” can only be maintained with the cooperation and dedication of a number of expert referees. The Editors would like to thank all those who have donated the hours necessary to review, evaluate and comment on manuscripts; their conscientious efforts have enabled the journal to maintain its tradition of excellence. We are grateful to the following reviewers for their contributions during 2023.

**C**

Chan Te‐Fu

Chang Chih‐Peng

Chang Chuan‐Hsin

Chang Hsueh‐Wei

Chang Kee‐Lung

Chang Kung‐Chao

Chang Long‐Sen

Chang Po‐Chih

Chau Gar‐Yang

Chen Chien‐Hung

Chen Chien‐Lin

Chen I‐Chen

Chen Jin‐Chung

Chen Ming‐Jen

Chen Pai‐Sheng

Chen Rong‐Fu

Chen Shih‐Jen

Chen Shiou‐Lan

Chen Yen‐Hsu

Chen Yih‐Fung

Chen Yuk‐Kwan

Cheng Hui‐Chen

Cheng Hung‐Chi

Cheng Tsung‐Lin

Chiu Yen‐Cheng

Cho Shih‐Feng

Choi Dong‐Young

Chou Cheng‐Yang

Chou Shah‐Hwa

Chu Chia‐Yu

Chu Hou‐Wei

Chuang Cheng‐Keng

Chuang Jing‐Jing

Chuang Shuang‐En

Chung Ching‐Hu

**D**

Du Je‐Kang

**F**

Fan Hsiu‐Lung

Francesco Facchinetti

Friedrich Christoph

**G**

Gean Po‐Wu

Guh Jih‐Hwa

**H**

Ho Shung‐Tai

Hsiao George

Hsiao Hui‐Pin

Hsieh Tsai‐Yuan

Hsieh Tusty‐Jiuan

Hsu Chung‐Yao

Hsu Han‐Jen

Hsu Jong‐Hau

Hsu Shih‐Hsien

Hsu Wen‐Hung

Hsu Ya‐Ling

Hu Stephen Chu‐Sung

Huang A‐Mei

Huang Ching‐Wen

Huang Chin‐Wei

Huang Chun‐Jen

Huang Chun‐Ming

Huang Jee‐Fu

Huang Mao‐Hsiung

Huang Ming‐Yii

Huang Shang‐En

Huang Shiang‐Fu

Huang Shu‐Pin

Huang Yu‐Tung

Hung Chao‐Hung

Hung Chi‐Feng

Hung Jan‐Jong

Hung Liang‐Yi

Hung Wei‐Wen

Hung Ying‐Chung

**J**

Jong Gwo‐Ping

**K**

Kaido Toshimi

Kanda Naoko

Kuo Kung‐Kai

**L**

Lan Cheng‐Che

Lan Sheng‐Hui

Lee Chaw‐Ning

Lee Che‐Hsin

Lee Chen‐Chen

Lee Chia‐Yang

Lee Chieh‐Ming

Lee Chien‐Hsing

Lee Ching‐Hao

Lee Chung‐Ta

Lee Hsiang‐Chun

Lee I‐Cheng

Lee Kuei‐Chuan

Lee Mei‐Hsuan

Lee Ming‐Fen

Lee Sheng‐Yu

Lee Woan‐Ruoh

Lee Yen‐Mei

Lee Yi‐Chen

Lee Ying‐Ray

Li Chien‐Feng

Li Ruei‐Nian

Li Wei‐Ming

Lieu Ann‐Shung

Lieu Hsiao‐Sheng

Lieu Yi‐Chang

Lin Chang‐Shen

Lin Chao‐Wen

Lin Cheau‐Feng

Lin Chi‐Chien

Lin Chien‐Ju

Lin Chih‐Wen

Lin Chun‐Yu

Lin Fan‐Li

Lin Hui‐Ching

Lin Jiun‐Nong

Lin Kun‐Der

Lin Ming‐Hong

Lin Shu‐Fu

Liu Yu‐Ping

Long Cheng‐Yu

Lu Da‐Wen

Lu I‐Cheng

**M**

Ma Zhong‐Liang

Miyagaki Tomomitsu

Muniswami Durai Murugan

**N**

Nadeem Ahmed

**P**

Pang See‐Tong

Peltomaeki Paeivi

**Q**

Qi Xiao‐Wei

**S**

Shi Hon‐Yi

Shiah Shine‐Gwo

Shih Yao‐Hsiang

Shiue Yow‐Ling

Su Chun‐Li

Su Yu‐Feng

Suen Jau‐Ling

Sun Hung‐Yu

Sun Jui‐Sheng

**T**

Tsai Eing‐Mei

Tsai Rong‐Kung

Tsai Siao Ping

Tsai Yi‐Chun

Tsai Yi‐Shan

Tseng Ping‐Huei

Tseng Sung‐Pin

Tseng Yu‐Hsin

**W**

Wang Chih‐Yuan

Wang Hui‐Chun

Wang Hui‐Min David

Wang Jing‐Houng

Wang Jiunn‐Wei

Wang Sheng‐Fan

Wang Shu‐Chi

Wang Su‐Jane

Wang Yao‐Kuang

Wang Yi‐Ching

Wu Shang‐Ju

Wu Chew‐Wun

Wu Chin‐Chin

Wu I‐Chen

Wu Yi‐Chia

Wu Zhi‐Fu

**X**

Xie Rong

**Y**

Yang Chih‐Jen

Yang Yu‐Chiao

Yeh Jen‐Hao

Yeh Jwu‐Lai

Yen Chia‐Hung

Yen Jiin‐Cherng

Yokoyama Kazushige

Yu Sebastian

Yuan Shyng‐Shiou

**Z**

Zhao Hao‐Sen

